# Volvulus Irritating the Myocardium: A Case Report

**DOI:** 10.7759/cureus.35256

**Published:** 2023-02-21

**Authors:** Elizabeth M Huycke, Suzanna S Tom, Alya Wezza, Gavin C Barr

**Affiliations:** 1 Department of Emergency and Hospital Medicine, University of South Florida Morsani College of Medicine/Lehigh Valley Health Network Campus, Allentown, USA

**Keywords:** tachyarrhythmia, ventricular arrhythmia, myocardium, organoaxial volvulus, tachycardia, gastric volvulus

## Abstract

Gastric volvulus is a rare condition that may present with various symptoms and may occur as an acute or chronic condition. Signs and symptoms may include nausea, vomiting, abdominal pain, and chest pain. It is imperative to recognize acute gastric volvulus in a timely fashion, since a delay in diagnosis may result in foregut obstruction and increased risk of strangulation, if not recognized and treated promptly. Additionally, secondary complications that are equally life-threatening, such as cardiac arrhythmias, can occur. For this very reason, it is important to highlight gastric volvulus as a possibility when developing a differential diagnosis in patients complaining of abdominal pain. This case report describes a 73-year-old female with no past cardiac risk factors, who presented to the emergency department (ED) with symptoms of supraventricular tachycardia (SVT), intermittent diarrhea, and nausea per emergency medical services (EMS). Upon EMS arrival at the patient’s home, her heart rate was 210 beats per minute (bpm). Despite her condition appearing to result from a cardiac condition, imaging studies found a large hiatal hernia through which the stomach had displaced. The patient’s stomach had distended, forming a volvulus and placing pressure on thoracic organs. This case highlights a rare but potentially life-threatening cardiac arrhythmia associated with gastric volvulus.

## Introduction

Gastric volvulus is defined as the abnormal rotation of all or part of the stomach around one of its axes [1]. Acute gastric volvulus is a serious condition that may require emergent laparotomy and may be combined with a thoracotomy in some cases [2]. It commonly presents with Borchardt’s triad (chest pain, severe emesis, and inability to pass a nasogastric (NG) tube) [3]. In most published case reports, Borchardt’s triad is the primary presentation leading to the discovery of gastric volvulus [4]. It is common for gastric volvulus to occur secondary to a hiatal hernia, and they can occur at any age [4,5]. Complications of volvulus include foregut obstruction and increased risk of strangulation, which could lead to a risk for necrosis, perforation, and hypovolemic shock, with mortality rates ranging from 30% to 50% [4]. It is important to recognize volvulus as a possible differential diagnosis in patients with chest pain because chest pain is the most common chief complaint seen in the emergency department (ED) [6]. This case report highlights the importance of identifying acute-onset gastric volvulus and illustrates an unusually associated cardiac complication.

## Case presentation

**Figure 1:**
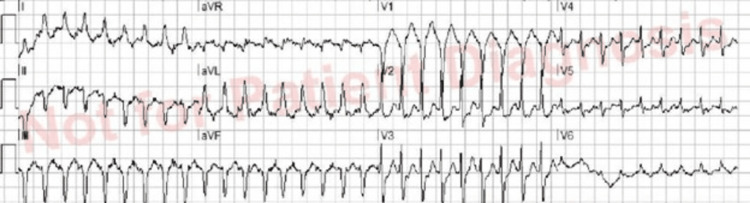
Prehospital ECG initially taken by EMS personnel prior to arrival at the ED. The patient’s heart rate was 210 bpm and showed signs of anterior septal and lateral ST-T abnormality consistent with myocardial ischemia. ECG: electrocardiogram, EMS: emergency medical services, ED: emergency department, bpm: beats per minute

A 73-year-old female with a past medical history significant for multiple sclerosis (MS) and urinary retention with a chronic indwelling Foley catheter presented to the emergency department (ED) with significant tachyarrhythmias per emergency medical services (EMS). The patient lived in a nursing home where her symptoms began. Her symptoms started two weeks prior to presentation and consisted of intermittent diarrhea and nausea without vomiting. According to the EMS report, they were called to the nursing home due to the patient experiencing shortness of breath (SOB) for approximately 10 minutes. When they arrived at the nursing home, the patient’s pulse was 210 beats per minute (bpm) with ECG findings consistent with myocardial ischemia (Figure 1). They promptly administered 150 milligrams (mg) of amiodarone per regional EMS protocol intravenously (IV) placed in the field to treat the patient’s tachyarrhythmia. However, after the administration of the amiodarone, the patient became hypotensive, and her condition worsened with no discernible effect on her rhythm.

**Figure 2:**
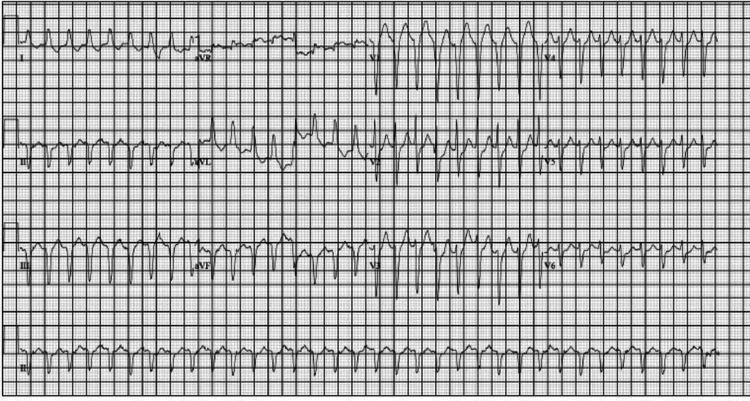
ECG of the patient upon arrival to the ED showing supraventricular tachycardia with a rate of 200 bpm. ECG: electrocardiogram, ED: emergency department, bpm: beats per minute

**Figure 3:**
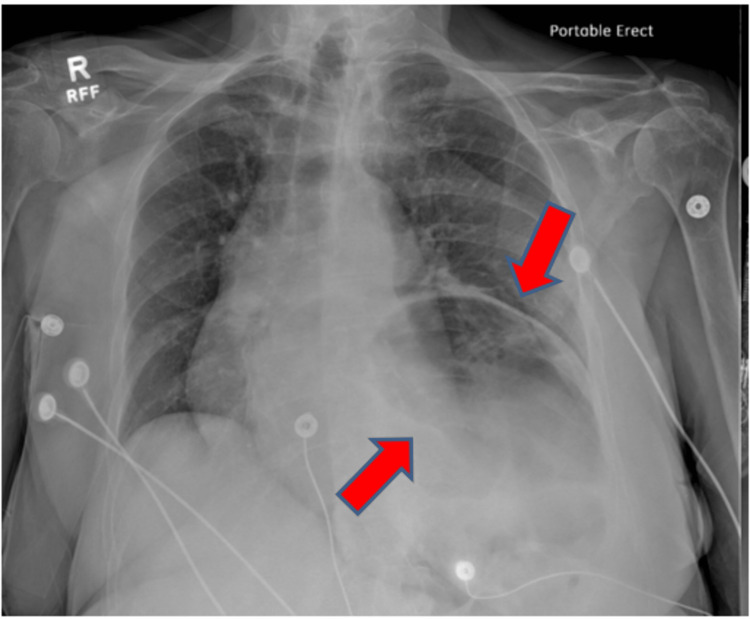
CXR showing elevated L hemidiaphragm with probable gas-distended splenic flexure, left basilar compressive atelectasis, and mild cardiomegaly (red arrows). CXR: chest X-ray, L: left

On arrival at the ED, the patient was tachypneic with an oxygen saturation of 94% on room air, profusely diaphoretic, and was still tachycardic with a heart rate of over 200 bpm (Figure 2). Her initial laboratory results upon arrival were as follows: blood pressure (BP) of 105/79 mmHg, pulse of 108 bpm, temperature of 36.4°C, and respiration of 20 breaths per minute (bpm). Her initial venous blood gas included pH 7.21 (normal values: 7.31-7.41), partial pressure of carbon dioxide (pCO_2_) of 24 mmHg (normal values: 41-51 mmHg), partial pressure of oxygen (pO_2_) of 74 mmHg (normal values: 30-40 mmHg), bicarbonate (HCO_3_) of 9 mEq/L (normal values: 23-29 mEq/L), oxygen saturation (spO_2_) of 91% (normal values: >75%), and base deficit of 16.7 (normal values: 0-6). These laboratory findings were interpreted as indicative of respiratory acidosis, which was concerning for a septic source, so she was started on 1 g of intravenous vancomycin and 1 g of intravenous cefepime. Chest X-ray (CXR) showed an elevated left hemidiaphragm with probable gas-distended splenic flexure and mild cardiomegaly. Additionally, a large hiatal hernia in the mediastinum was causing a mediastinal shift (from an organoaxial gastric volvulus) (Figure 3). The patient had an elevated serum lactate of 5.3 mmol/L (normal values: 0.5-2.1 mmol/L) and leukocytosis of 16,000/cmm (normal values: 4-10,000/cmm). The patient also had elevated red blood cell count (RBC) at 4.90 mill/cmm (normal values: 3.70-4.70 mill/cmm). The rest of her complete blood count panel was unremarkable upon initial presentation. Blood cultures were negative for any bacterial growth.

**Figure 4:**
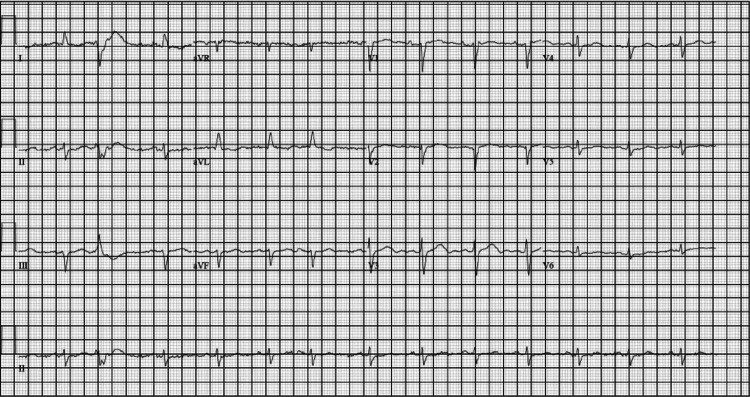
ECG of the patient showing normal sinus rhythm with occasional PVCs and PACs after several rounds of chemical and electrical cardioversion. ECG: electrocardiogram, PVCs: premature ventricular contractions, PACs: premature atrial contractions

In the ED, the patient was given 6 mg of adenosine, and she briefly converted to normal sinus rhythm (NSR) before developing supraventricular tachycardia (SVT). Subsequently, she was given an additional 12 mg of adenosine, which did not resolve her SVT. She was then synchronously cardioverted with 120 J and was immediately synchronously cardioverted again at 200 J, after which she converted to NSR (Figure 4). For hypotension, she was also given a total of 200 micrograms (µg) of phenylephrine in three push doses with no improvement. Her BP improved after being started on a norepinephrine drip at 12 µg/minute. After conversion to NSR, the patient no longer complained of SOB or diaphoresis.

**Figure 5:**
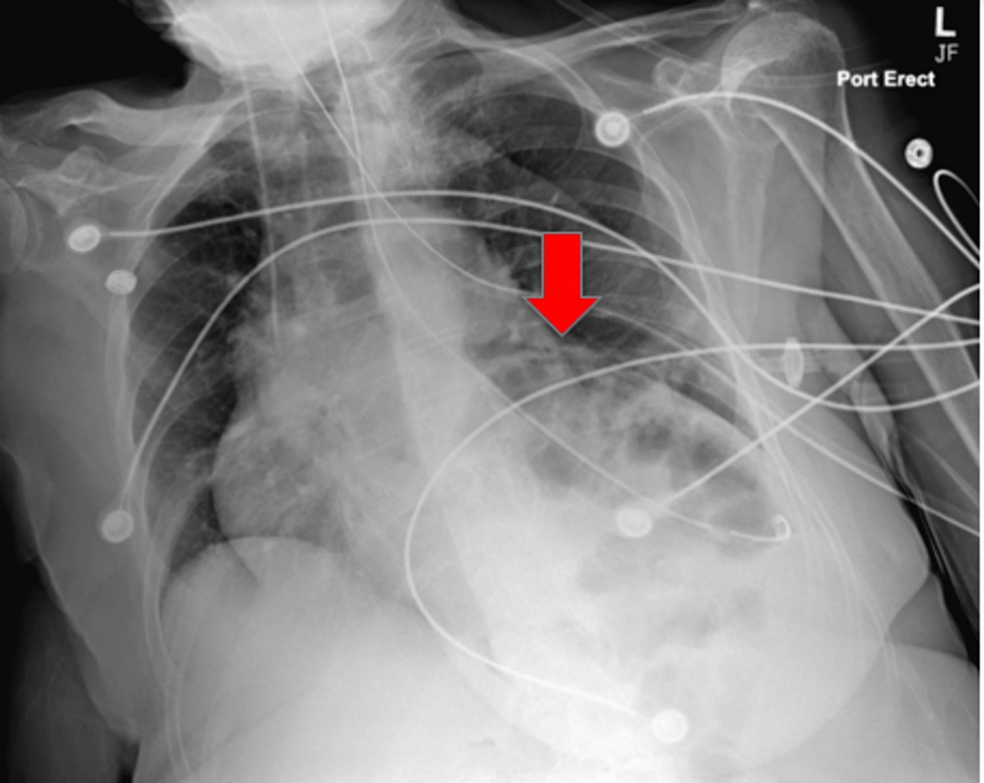
Post-EGD CXR shows persistent L hemidiaphragmatic defect extending into the L hemithorax with associated L basilar atelectasis (red arrows). EGD: esophagogastroduodenoscopy, CXR: chest X-ray, L: left

The stomach was distended, indicating possible gastric outlet obstruction. The patient was promptly admitted to the surgical service, and the gastrointestinal (GI) team performed an upper endoscopy without air insufflation to facilitate the placement of a nasogastric (NG) tube. The endoscopy identified a hyperemic gastric mucosa without overt necrotic features, and the interventionist indicated that the endoscope was successfully navigated to the duodenum using gentle water immersion. At the end of the procedure, the NG tube was successfully placed. A post-esophagogastroduodenoscopy (EGD) CXR was obtained to evaluate volvulus correction, which showed persistent left hemidiaphragmatic defect extending into the left hemithorax with associated left basilar atelectasis (Figure 5).

**Figure 6:**
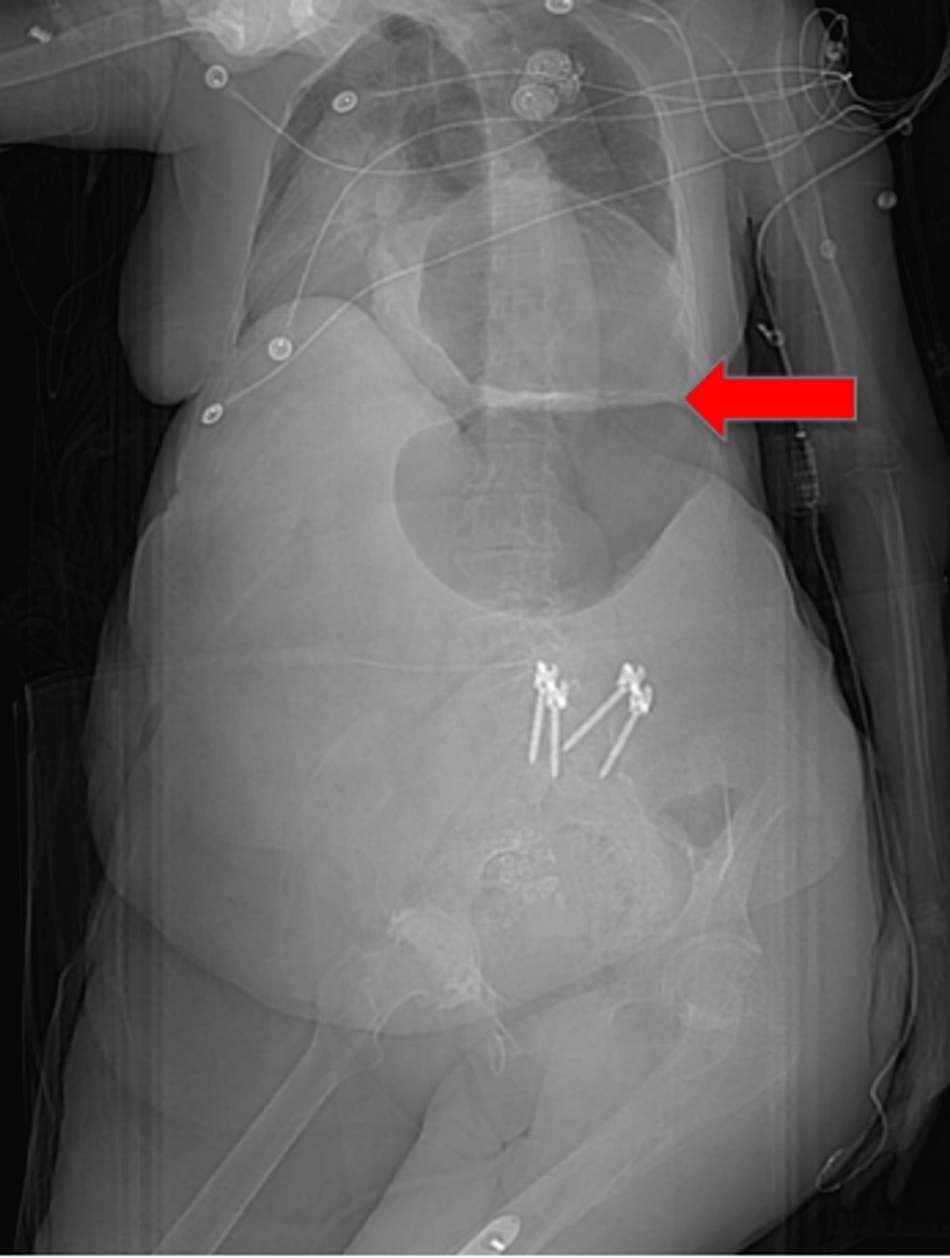
CT of the chest, abdomen, and pelvis with IV contrast on the patient’s second admission. The figure shows a large hiatal hernia, with organoaxial volvulus and significant gastric distention, findings concerning for gastric outlet obstruction. CT: computed tomography, IV: intravenous

Since the resolution was unclear, the gastroenterologist planned to reevaluate the patient with an upper gastrointestinal (UGI) series of radiography after a period of NG decompression. A GI fluoroscopy was obtained two days after the patient’s initial presentation and showed no obstruction at the gastric outlet. She tolerated the advancement of her diet and had no further arrhythmia after the decompression of her stomach. She was discharged 19 days after her initial presentation with a planned outpatient hernia repair. Unfortunately, she returned to the ED again just six days after discharge with similar symptoms as when she initially presented such as intermittent diarrhea and nausea with no tachyarrhythmias present. She also reported abdominal pain without fevers and anorexia, with a witnessed large maroon bowel movement and coffee ground emesis. The CT again showed volvulus of the herniated stomach with significant distention, concerning for gastric outlet obstruction (Figure 6).

Following the placement of an NG tube, the patient’s condition improved enough to suggest that the volvulus was detorsed, and GI forwent EGD in favor of hernia repair. The following day, the patient was cleared for surgery by cardiology, and three days later, she underwent laparoscopic repair of the incarcerated hiatal hernia with gastropexy. The patient’s recovery post-surgery was not complicated, and she was discharged home five days later.

## Discussion

Volvuli can arise from the obstruction of several segments of the GI tract or its displacement through a hernia. The risk of cardiac arrhythmias and “sudden cardiac death” in patients with sigmoid volvulus has been reported [7]. Unlike gastric volvulus, which is primarily associated with the stomach, sigmoid volvulus is found in the large intestine [8]. There is relatively sparse research on both volvulus conditions, and they may present with similar symptoms and side effects. Therefore, it is important to differentiate between which volvulus is present to accurately treat the patient and improve care coordination and communication to ultimately improve outcomes [6].

Detection and identification of a volvulus as the underlying condition in a patient can be difficult, as symptoms are typically nonspecific [3]. Borchardt’s triad can reliably indicate the presence of gastric volvulus, although it is uncommon to attempt the insertion of a nasogastric tube in the ED setting, especially if the patient is experiencing symptoms that require emergent intervention [3]. Therefore, imaging studies must be relied upon to make a prompt diagnosis and expedite treatment in the ED.

Our case highlights a rare but potentially lethal cardiac arrhythmia secondary to a gastric volvulus and hiatal hernia. Tachyarrhythmias with unstable vital signs, likely from irritation of the myocardium, can be an immediate life-threatening problem. The onset of the cardiac arrhythmia and the cognitive approach to its management may easily have distracted a clinician from further evaluation. Others have reported the possible cardiac side effects that may present in the case of a gastric volvulus [9]. Cardiac arrhythmias and sinus tachycardia have been reported to be associated with volvulus in previous case studies [10]. Despite having been previously reported, the rarity of cardiac arrhythmias resulting from a hiatal hernia and gastric volvulus makes its diagnosis somewhat difficult.

Gastric volvulus treatment strategies range from supportive care to operative interventions. When a chronic gastric volvulus manifests in a non-life-threatening manner, conservative treatment approaches are preferred [11]. These minimally invasive strategies include monitoring the patient, placing them prone to decompress the abdomen, and providing supportive care such as fluid resuscitation [10]. By decompressing and observing non-emergent cases of gastric volvulus, it is possible that the condition will resolve itself with time, and the stomach returns to its normal shape. However, more severe cases require more direct interventions.

Laparoscopic surgical procedures are the preferred method of intervention in progressive or acute cases [12]. Depending on the severity of the volvulus, patients can typically be treated with excision of the hernia sac, an anti-reflux procedure, and laparoscopic placement of a gastronomy tube [12]. The gastronomy tube helps secure the stomach in its proper position and prevents remigration into the thoracic cavity. In cases where full-thickness necrosis is present, a gastric resection is necessary [12]. While this approach has the highest resolution rate of the volvulus, less invasive procedures should be attempted first, when possible.

## Conclusions

This case report serves as an example of myocardial irritation resulting in cardiac arrhythmia secondary to a gastric volvulus. As such, gastric volvulus should be considered in patients who present with tachyarrhythmia without a previously diagnosed heart condition, particularly when the patient has imaging demonstrating a hiatal hernia with or without gastric herniation or epigastric gas bubble. In such cases, cardiac arrhythmias are likely secondary to volvulus and highlight the importance of including volvulus when developing a differential diagnosis.
